# Correction: St. Jude Survivorship Portal: Sharing and Analyzing Large Clinical and Genomic Datasets from Pediatric Cancer Survivors

**DOI:** 10.1158/2159-8290.CD-24-1432

**Published:** 2024-12-02

**Authors:** Gavriel Y. Matt, Edgar Sioson, Kyla Shelton, Jian Wang, Congyu Lu, Airen Zaldivar Peraza, Karishma Gangwani, Robin Paul, Colleen Reilly, Aleksandar Acić, Qi Liu, Stephanie R. Sandor, Clay McLeod, Jaimin Patel, Fan Wang, Cindy Im, Zhaoming Wang, Yadav Sapkota, Carmen L. Wilson, Nickhill Bhakta, Kirsten K. Ness, Gregory T. Armstrong, Melissa M. Hudson, Leslie L. Robison, Jinghui Zhang, Yutaka Yasui, Xin Zhou

In the original version of this article (1), several labels in Fig. 1A were inadvertently mislabeled as “mol/L” by the compositor during production of the paper and should instead read “M” to indicate million. Figure 1A has been corrected in the latest online HTML and PDF versions of the article. The publisher regrets the error.
